# A Liberal Offer

**Published:** 1886-10-20

**Authors:** R. C. Word

**Affiliations:** Managing Editor


### EDITORIALS AND MISCELLANEOUS.

MANAGING EDITOR.

### A LIBERAL OFFER.

Our subscribers could, with very little trouble to themselves, double our subscription list between this and the time of our next issue. To do this it would only be necessary for each one to send us one new subscriber. Will our friends not do this for us? It would help us to improve and enlarge the RECORD, and thus result in benefit to all. But we will not ask, even this, of you for nothing. We will offer a very liberal Premium. It is this: THE WESTERN PLOWMAN is a handsome, vigorous, and practical twenty-eight page monthly, devoted to the best interests of the Home, Farm and Family. Not sensational, but aggressive, fearless, and full of Western vim and snap. Its agricultural information will be fresh from the fields, and reliable. Its home reading matter will be pure and instructive, but too highly seasoned with wit, humor, pathos, and spirit to be dull. It has a rod in pickle for a million social follies, and it will be wielded vigorously, no matter whom it may strike. No farmer can afford to be without it. The housewife needs it for the practical information contained in its household department. The boys and girls will be benefitted and instructed by the live, wide-awake articles, sketches, puzzles, poetry, wit and humor which enliven its pages. It is a household paper, containing no trash, no exaggerated pictures of life, no sensational "news," but a careful record of all that is intended to make life better than it is. It contains more matter and of a better character than most of the two dollar monthlies, but we offer this literary feast AS A PREMIUM, FOR NOTHING, to every reader who will send us one new subscriber and enclosing $2, the cash price of the RECORD for one year.

R. C. WORD, M. D., Managing Editor.

